# A Millifluidic Chamber for Controlled Shear Stress Testing: Application to Microbial Cultures

**DOI:** 10.1007/s10439-023-03361-4

**Published:** 2023-09-15

**Authors:** Francesco Biagini, Ermes Botte, Marco Calvigioni, Carmelo De Maria, Diletta Mazzantini, Francesco Celandroni, Emilia Ghelardi, Giovanni Vozzi

**Affiliations:** 1 Research Center “E. Piaggio”, University of Pisa, Largo L. Lazzarino 1, 56122 Pisa, Italy; 2 Department of Information Engineering, University of Pisa, Via G. Caruso 16, 56122 Pisa, Italy; 3 Department of Translational Research and New Technologies in Medicine and Surgery, University of Pisa, Via San Zeno 35, 56123 Pisa, Italy

**Keywords:** Cell adhesion, Flow-exposed cell cultures, Computational fluid dynamics, In vitro models, Human gut microbiota

## Abstract

In vitro platforms such as bioreactors and microfluidic devices are commonly designed to engineer tissue models as well as to replicate the crosstalk between cells and microorganisms hosted in the human body. These systems promote nutrient supply and waste removal through culture medium recirculation; consequently, they intrinsically expose cellular structures to shear stress, be it a desired mechanical stimulus to drive the cell fate or a potential inhibitor for the model maturation. Assessing the impact of shear stress on cellular or microbial cultures thus represents a crucial step to define proper environmental conditions for in vitro models. In this light, the aim of this study was to develop a millifluidic device enabling to generate fully controlled shear stress profiles for quantitatively probing its influence on tissue or bacterial models, overcoming the limitations of previous reports proposing similar devices. Relying on this millifluidic tool, we present a systematic methodology to test how adherent cellular structures react to shear forces, which was applied to the case of microbial biofilms as a proof of concept. The results obtained suggest our approach as a suitable testbench to evaluate culture conditions in terms of shear stress faced by cells or microorganisms.

**Graphical Abstract:**

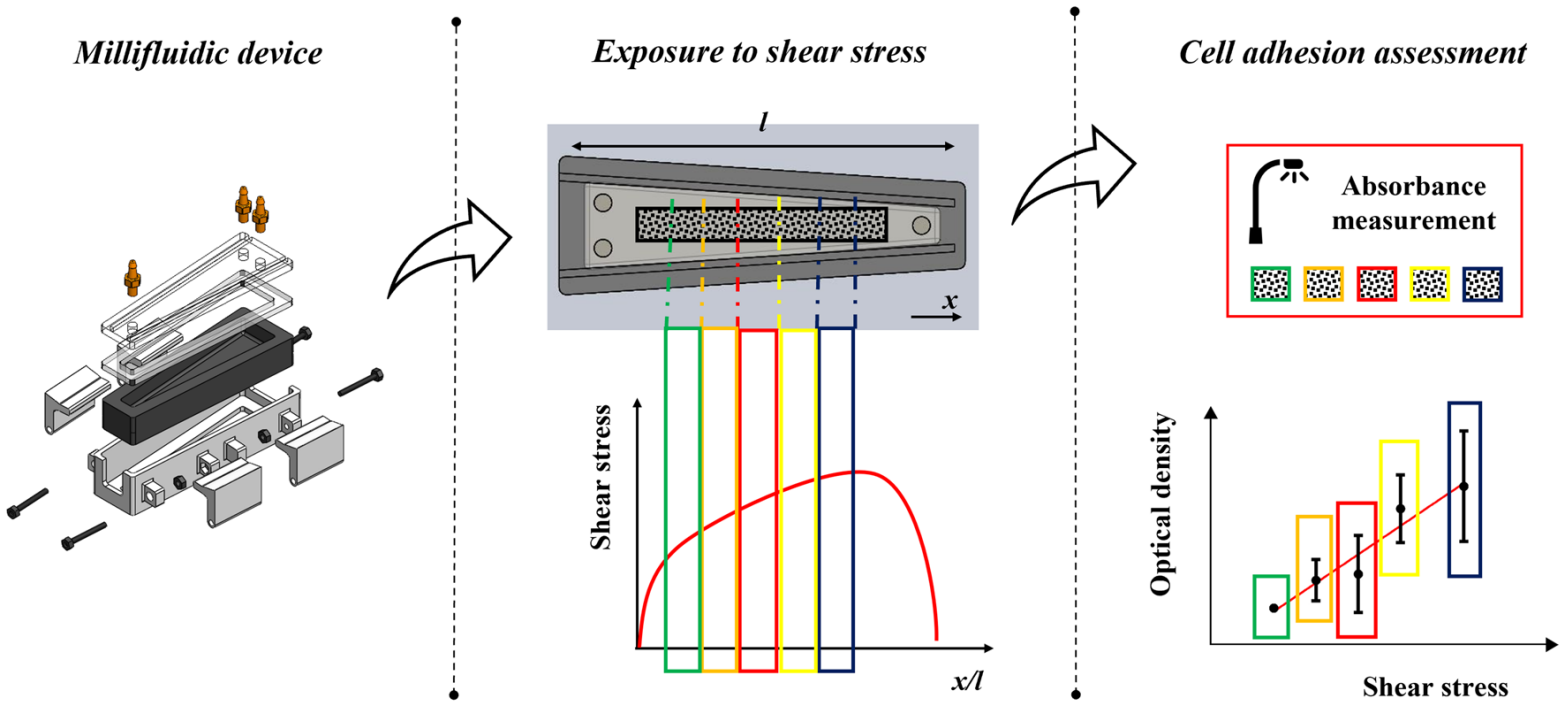

## Introduction

Shear stress represents one of the most common mechanical stimuli which cells—be they eukaryotic cells or microorganisms of the gut flora—are physiologically exposed to in the human body [1, 2]. In recent decades, its use to improve the relevance of specific tissue models in vitro has raised great interest [3, 4]. To this end, fluidic culture systems (i.e., involving the recirculation of culture media) have been increasingly employed either to enhance cell proliferation through convective nutrient supply and waste removal or to purposely apply viscous forces [4, 5]. For instance, Lindner et al. demonstrated that, if compared to static culture conditions, shear stress between 0.2 and 0.8 mPa promotes the self-arrangement of intestinal epithelial cells in three-dimensional (3D) villi-like structures [6]. Similarly, shear forces associated with fluid flow were shown as a necessary condition to induce phenotypic traits of endothelial cells, such as coherent orientation, non-thrombogenicity and healing potential [7, 5]. On the other hand, several works dealt with the design of low-shear stress bioreactors for the development of tissue constructs embedding shear-sensitive cells (e.g., hepatocytes) [8, 4]. In these cases, the aim was to set a suitable trade-off between the extent of viscous forces (shear stresses of few µPa) and the advantages related to convective mass transport.

Unlike such extensive literature on the impact of shear stress on eukaryotic cells, a few studies report on how it affects microbial cultures, although fluidic systems are also widely used for recapitulating the microenvironment of the human gut and even the crosstalk between cells and bacteria [9–11]. The ability of microorganisms to adhere on the culture substrate and form a stable biofilm (i.e., an aggregation of bacteria arranged to create 3D structures by producing extracellular matrix) can be impaired or promoted by flow exposure, depending on both the physio-chemical properties of the cell membrane and the eventual presence of external appendages (e.g., pili, flagella) [12]. In this regard, some studies highlighted that biofilm-substrate interactions mediated by specific appendages (such as type I fimbriae [13]) are strengthened by shear forces. Hence, an increasing shear stress reduces microbial detachment under flow, while weaker adhesion was observed at low shear stress levels due to rolling effects [14, 15]. Though, besides adhesion, Thomen et al. demonstrated the existence of a threshold mechanism, according to which shear stresses higher than 10 mPa inhibit the proliferation of just inoculated *Escherichia coli* [16]. Similar dynamics on a higher scale of shear forces were noticed regarding the viability of bacteria in *E. coli* (viability threshold of 1.29 kPa, even membrane lysis for stress magnitudes higher than 1.81 kPa) and *Saccharomyces cerevisiae* specimens (viability threshold of 1.25 kPa) [17].

Irrespective of the biological character of cells, all the mentioned literature relied on simple fluidic devices which face some limitations. In their series of papers, Lange et al. tested the correlation of *E. coli* and *S. cerevisiae* viability with the extent of the applied shear stress based on a capillary tube system with known cross-section area [18]. Given the inoculum density, they preliminarily characterized the rheology of microbial suspensions and subsequently estimated shear tensions at the tube wall assuming a Poiseuille’s velocity profile [17]. Despite the easiness of such experimental set up, the approach suffers of two main drawbacks: (*i*) bacteria could cluster under flow [19] and thus significantly deviate the rheological response of the suspension (i.e., the bigger the clusters, the further from purely Newtonian the behaviour), affecting its viscosity estimation; (*ii*) bacteria are implicitly assumed to flow in the vicinity of the boundary, neglecting that they might undergo different shear stress levels from each other or even at subsequent circulation instances through the capillary as a function of their radial position in the channel. Overall, these analytical simplifications may alter the identified threshold for viability, which is likely to be overestimated. For adherent mammalian cells, the reported examples exploited soft bioreactors moulded in biocompatible poly-dimethyl-siloxane (PDMS) [4, 5]. Here, cells plated onto the bottom wall of the culture chamber perceive a homogeneous shear stimulus on the scale of µPa, which can be tuned through the inlet flow rate on the basis of preliminary computational fluid dynamics (CFD) simulations [5]. However, this in silico modelling does not consider structural deformations induced to ensure the hydraulic sealing of the system, altering the cross-section geometry and, hence, the applied viscous stress. Furthermore, given the inlet flow rate, all described approaches expose cells or microorganisms to the same, constant level of shear stress, so that very high flows would be required to achieve high stresses. This might be a challenge, due to sealing issues related to the increasing hydrostatic pressure as well as to the potential onset of vorticity phenomena [20]. A first study on a fluidic device able to generate linearly increasing shear stress profiles up to 4 Pa is that reported by Usami et al. [21]. In this case, the use of glass plates ensures the retention of geometrical features of the chamber, but shear stress estimations rely on simple analytical calculations assuming a 2D Poiseuille’s flow.

In this context, our study aimed at providing a more reliable methodology to probe cell response to shear stress, in the perspective of designing in vitro platforms for engineered tissue and microbial models. Specifically, we first exploited CFD to design a millifluidic chamber allowing to expose biological samples to controlled shear stress profiles, overcoming typical issues of state-of-art devices in the field. A prototype of the chamber was fabricated, and its performance verified. Then, the device was included as part of an experimental pipeline purposely developed to evaluate the impact of viscous forces on adherent cellular structures. As a proof of concept, this methodology was finally implemented to assess microbial biofilm formation under controlled shear stress magnitudes for single bacterial species (namely, *E. coli* and *Enterococcus faecalis*) and the complete human gut microbiota.

## Materials and Methods

CFD models were implemented by means of finite element (FE) methods using Comsol Multiphysics (version 5.3, COMSOL AB, Stockholm, Sweden), while the computer aided design (CAD) and manufacturing (CAM) of single components of the device were performed in Autodesk Fusion 360 (Autodesk, California, USA). GraphPad Prism (version 6, GraphPad Prism Software, California, USA) was used for all statistical analyses.

### The Millifluidic Chamber

#### Conceptual Design

**Fig. 1:**
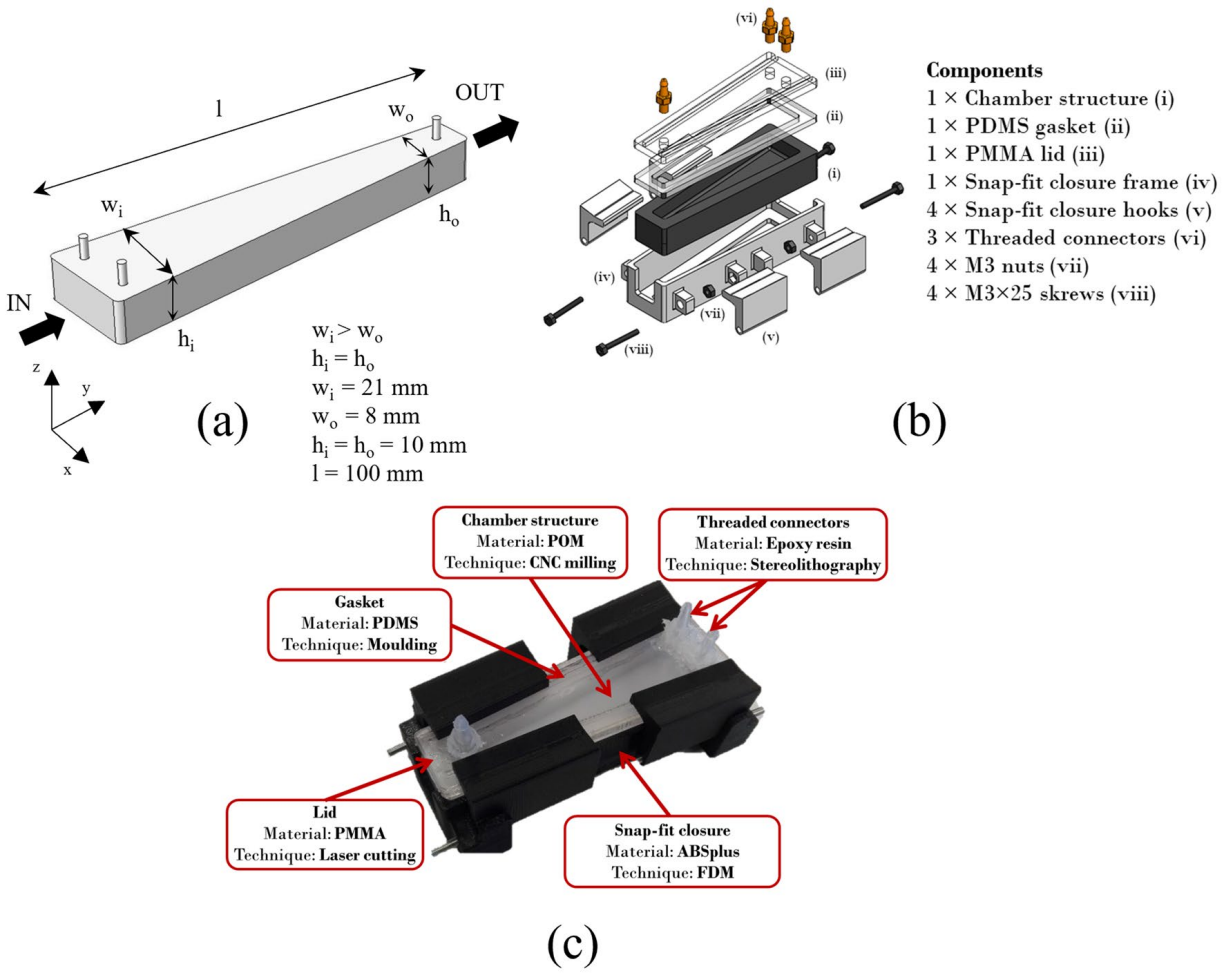
Design and fabrication of the millifluidic chamber for the generation of controlled shear stress profiles. **a** Conceptual design of the chamber, used for FE analysis. **b** Exploded view of the millifluidic chamber components. The device is composed of the chamber structure (i), a flat gasket (ii), a transparent lid (iii), the snap-fit closure frame (iv), four hooks for tightening the snap-fit closure (v), three custom threaded connectors to connect the chamber to external hydraulic circuitry during experiments (vi) and four M3 nuts with threaded bolts (vii–viii) to fasten the hooks to the frame. **c** A prototype of the millifluidic chamber. Materials and techniques used for the fabrication of each component are specified

The millifluidic chamber was designed to generate spatial gradients of shear stress at the bottom wall of the device, where adherent cellular structures can be placed and hence exposed to controlled stress levels. In this light, we chose a geometry consisting of a pyramid trunk with constant height (*h* = 10 mm, Fig. 1a) and width (*w*) linearly decreasing from 21 to 8 mm (Fig. 1a) along the longitudinal axis (i.e., the *y* axis in Fig. 1a), to get a cross-section area which in turn decreases in the direction parallel to that axis (i.e., the principal direction of the flow). According to the Poiseuille's law for a channel having rectangular cross-section, this guarantees a positive longitudinal gradient of wall shear stress from the inlet to the outlet section, given the non-linear pressure drop along the channel. In this way, the cell response to a spatial range of stresses can be assessed setting a single flow rate. Moreover, the monotonically increasing shear stress profile induced by the chosen design hinders that potentially detached cells settle or even adhere closer to the outlet channel, avoiding undesired alterations of the biofilm spatial distribution. The flow was split in two entries (Fig. 1a) to reduce the velocity drop at the inlet section of the chamber and thus minimize vorticity, which could cause uncontrolled stress peaks on the biological specimen. In addition, both the inlet and outlet channels were designed to orthogonally pass through the upper wall of the device in order to facilitate the filling procedure and the removal of air bubbles.

#### Finite Element Analysis

A CFD model of the proposed design was run to numerically estimate the shear stress field at the bottom of the chamber as a function of the flow rate. Fig. 1a reports relevant dimensions of the simulated 3D geometry, which was directly imported as a .stl file from the CAD software. These geometric parameters were deemed as the most suitable for our purpose and consequently considered for the realization of the prototype (see the following sub-section). The model solved the Navier-Stokes equation for an incompressible fluid in conditions of laminar flow (“Navier-Stokes incompressible, laminar flow” module in Comsol), and a steady-state analysis parametrized with respect to the flow rate was performed.

The geometry consists of a single domain describing the circulating culture medium (i.e., RPMI 1640, see the sub-section C*ulture and exposure of microorganisms to flow*), whose rheological properties were set as domain conditions (density: *ρ* = 993 kg m^−3^; dynamic viscosity: *η* = 7 × 10^−4^ Pa s [8, 4]). In all simulations, given the dimensions of the system, the volume force associated with gravity was neglected. Regarding boundary conditions, two different flow rates (20 and 40 mL min^−1^) were equally set at each inlet channel (hence, the overall flow rate in the chamber was two times that at the inlet), while the outlet was taken as a reference for the pressure field. On all the other boundaries, a "no-slip" condition was considered, which implies a null fluid velocity at these walls. Once the geometry had been discretized through the generation of a fine mesh controlled by the physics (673623 elements with a minimum size of 0.193 mm), it was possible to solve the models calculating the velocity and pressure fields, and thus the wall shear stress at the bottom, for each flow rate value.

#### Fabrication of the Millifluidic Chamber Components

Figure 1b shows the different components used for the prototyping of the device. In particular, to facilitate the hydraulic sealing and avoid significant deformations when tightening the closure, the millifluidic chamber was fabricated in three main components: a rigid chamber structure, a silicone gasket allowing the effective sealing of the device and a transparent lid for closing and visual control during experiments. To make the medium inlet and outlet passing through the lid, a threaded coupling was designed between customized hydraulic connectors and appropriate holes.

The prototype was fabricated using different materials as well as additive and subtractive manufacturing techniques, as summarized in Fig. 1c. The chamber structure was made of poly-oxymethylene (POM), using a 3-axis CNC milling machine (SRM-20, Roland DG) equipped with a flathead milling cutter having diameter of 3 mm. POM is biocompatible, rigid and non-porous [22], ensuring the geometrical stability of the device, and thus a reliable correspondence between the predicted and experimental shear stress profile, unlike systems based on PDMS [23]. The lid was fabricated by laser cutting, using a Trotec Speedy100 (Trotec, Marchtrenk, Austria) machine to shape a 5 mm thick glassy poly-methyl-methacrylate (PMMA) sheet. PMMA is a biocompatible material, non-porous, and transparent. After the cutting phase, the component was completed by drilling the holes for the insertion of the inlet and outlet threaded connectors. The latter were 3D printed by stereolithography (Form 2, Formlabs, Massachusetts, USA) using a biocompatible epoxy resin (Dental SG Resin, Formlabs). The flat gasket was fabricated in PDMS (Sylgard-184, Dow Corning, Michigan, USA) with a monomer/initiator ratio of 10:1 w/w, following the producer’s protocol. To simplify the assembly and disassembly phases of the device as much as possible and guarantee a uniform tightening compression, we chose a 'snap-fit' closing system. The snap-fit closure was fabricated in ABSplus by fused deposition modelling (FDM) using a Fortus 250mc (Stratasys, Rehovot, Israel) industrial 3D printer. The hydraulic seal of the device when closed using the snap-fit system was assessed by applying a constant flow rate of 24 mL min^−1^ for 12 h. No leakage occurred during the test, confirming the effectiveness of the chosen closure.

### Proof-of-Concept-Application to Microbial Cultures

#### Microbial Strains, Fecal Microbiota and Preparation of the Culture Substrate

*E. coli* (ATCC 25922) and *E. faecalis* (ATCC 29212) were selected as reference strains, as they are typical commensals of the human gut microbiota. A healthy stool donor was selected in accordance with inclusion/exclusion criteria as previously described [24]. Faecal samples were collected and processed following the recent European guidelines for faecal microbiota transplantation [25], then stored at − 80 °C in 10% v/v glycerol [24] until use.

The culture substrate was obtained in the form of a rectangular strip of biocompatible polyester (Sigma-Aldrich) having dimensions 8 × 75 mm, to be longitudinally placed onto the bottom of the millifluidic chamber just before the beginning of the experiment. Based on the CFD model, the strip length was designed to match the useful length of the chamber (i.e., the region of the chamber where a nearly linear shear stress profile is generated and no vortices arise, see Fig. 3), while its width was chosen to ensure that the specimen undergoes an almost constant shear stress along the direction orthogonal to the primary flow. Before seeding, the strips were sterilized with a 70 % v/v ethanol solution and subsequently exposed to UV light for 15 min on both sides.

#### Culture and Exposure of Microorganisms to Flow

For each experiment, a volume of 600 µl of bacterial or faecal suspension was inoculated onto the culture substrate, placed within a Petri dish containing 15 mL of RPMI 1640 medium (Sigma Aldrich), referred to in the following as simply medium. To allow the development of a stable biofilm, microbial cultures were incubated for 24 h at 37 ºC in anaerobic conditions before testing. Hypoxia was induced in the culture environment using Oxoid AnaeroGen (Termo Fisher Scientific).

Then, the specimen was moved from the Petri dish to the millifluidic chamber and exposed to medium recirculation—and, hence, to the desired shear stress profile—for 8 h, keeping again hypoxic conditions through Oxoid AnaeroGen filters. The test was carried out at room temperature (RT, i.e., 25 ºC) under a laminar flow hood guaranteeing aseptic conditions, using a peristaltic pump (IPC, Ismatec) to create a closed hydraulic circuit which also included a culture medium reservoir and silicone tubes with an internal diameter of 2 mm. Overall, 30 mL of medium were circulating in the system. In parallel, a second specimen of the same type was maintained in static culture conditions (i.e., no fluid flow) within another prototype of the millifluidic chamber, integrated into the hydraulic circuit and filled with the same volume of RPMI 1640. This specimen was considered as a reference to evaluate the differential increase of biomass at the applied shear stress levels.

For both single bacterial strains as well as human gut microbiota samples, two different flow rates (20 mL min^−1^ and 40 mL min^−1^) were set at the inlet of the millifluidic chamber, and tests were carried out in triplicate per each.

##### Assessment of Differential Biofilm Adhesion and Correlation with the Applied Shear Stress Levels

After the exposure to flow, the adhered biomass on the culture substrate was quantified by means of a crystal violet assay for both the flow-exposed culture and the static control. Crystal violet stains both microorganisms and the extracellular matrix they synthesize [24, 26]. Its accumulation can be thus related to the development of the whole biofilm through proper absorbance measurements of stained specimen.

**Fig. 2:**
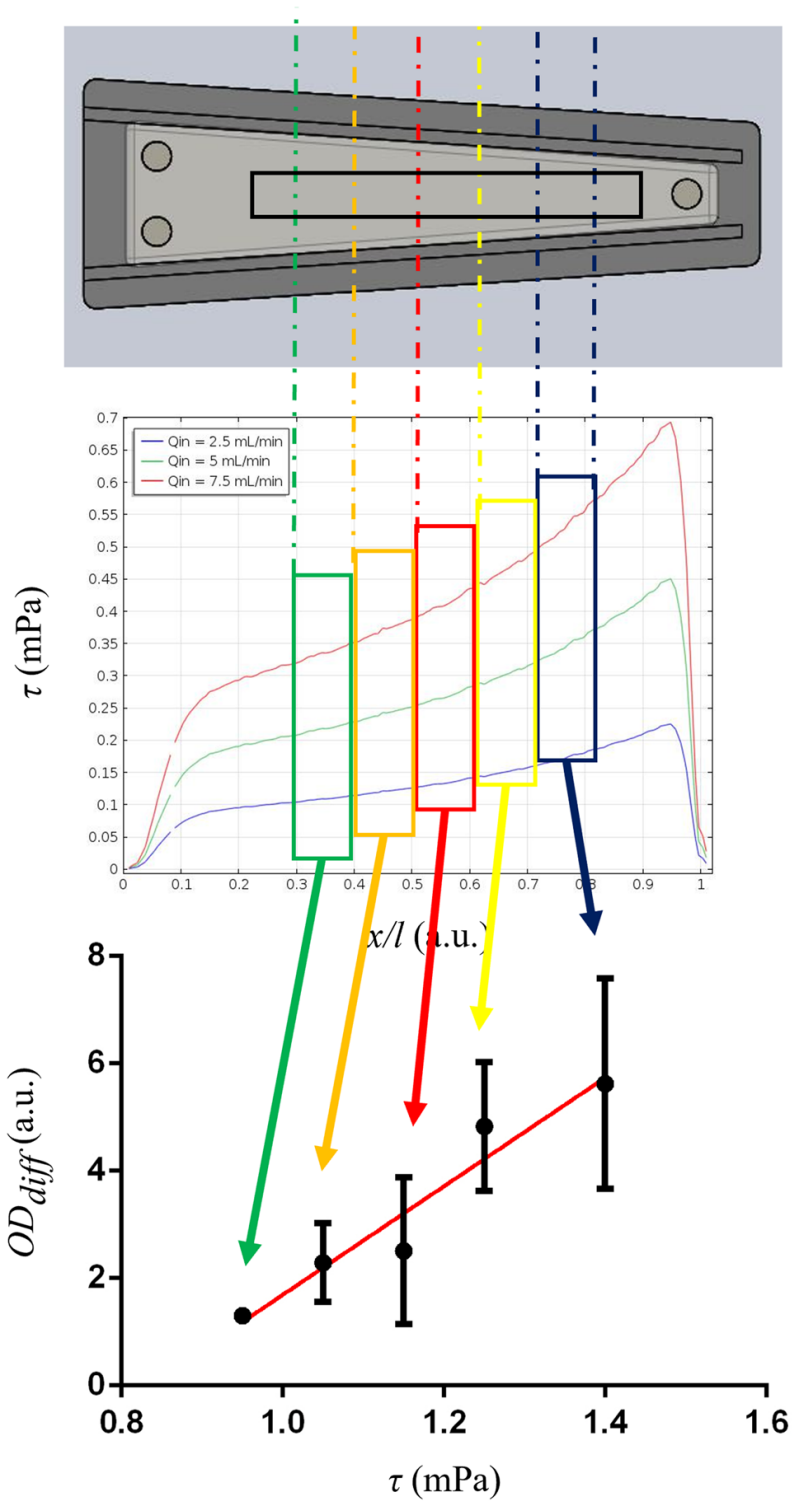
Rationale of the association between differential biofilm adhesion and applied shear stress. The longitudinal shear stress profile predicted by FE analysis for the specific flow rate is stepwise averaged according to the position of specimen ROIs during the experiment. Such average values are then associated with the differential absorbance measured for corresponding ROIs, and the statistical correlation of the two is evaluated

**Fig. 3:**
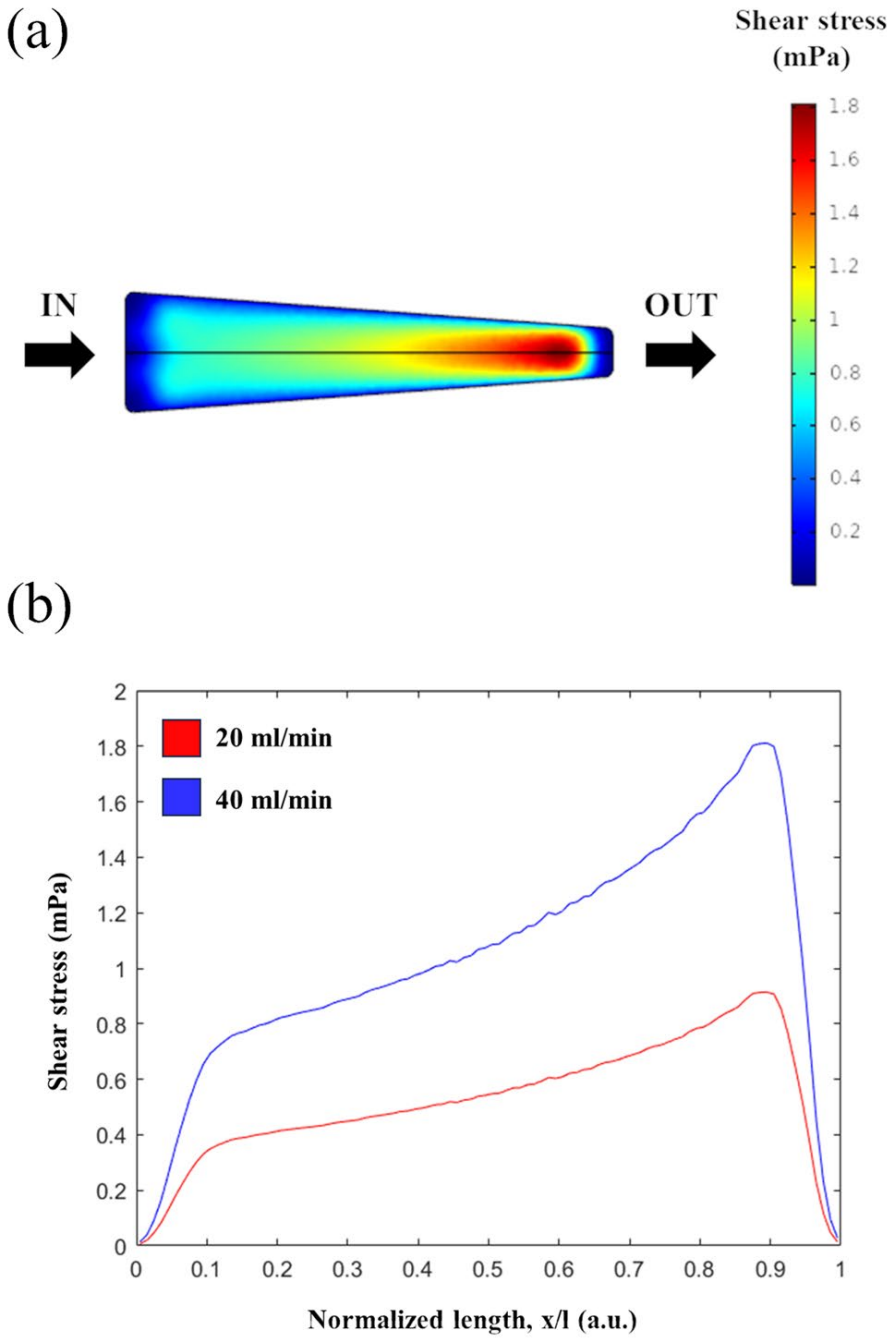
Shear stress field **a** on the bottom of the millifluidic chamber (*Q* = 40 mL min^−1^) and **b** along the middle longitudinal line in the flow direction parametrized with respect to the set flow rate

As first, the culture medium was gently removed from both millifluidic devices, and microbial samples were rinsed three times with PBS 1 × eliminate non-adherent planktonic microorganisms, which might have detached during the experiment and randomly settled onto the biofilm. 5 equally sized (i.e., 8 × 10 mm) regions of interests (ROIs) were cut from the middle part of polyester strips with adhered bacteria, discarding 7.5 mm from both the proximal and distal part with respect to the inlet of the device. This was in the light of avoiding regions which had potentially undergone shear stress peaks during the experiment (see Fig. 3). A volume of 200 µL of 0.1% w/v crystal violet (Carlo Erba, Italy) was used to stain microbial biofilms developed onto each ROI while placed within a 96-well plate for 30 min at RT [26]. Then, each sample was rinsed three times with deionized water to wash the non-internalized dye and subsequently covered with 200 µL of absolute ethanol for 15 min at RT to solubilize the crystal violet uptaken by bacteria. Aliquots of ethanol-crystal violet solution (200 μL) were transferred to a clean 96-well plate and their optical density at 570 nm (*OD570*, simply referred to in the following as *OD*) was measured by using a microplate reader (Biorad model 550, Biorad, USA) [27, 28]. Blank controls (i.e., without microorganisms) were also performed in triplicate according to the same protocol to correct for the intrinsic *OD* of polyester (*OD*_*bl*_). Given these measurements, the differential absorbance (*OD*_*diff*_) of each ROI was estimated as:

1 $${OD}_{diff}= \frac{{OD}_{flo}- {OD}_{bl}}{{OD}_{sta}- {OD}_{bl}}$$

where *OD*_*flo*_ and *OD*_*sta*_ are the *OD* measured for the flow-exposed culture and the static control, respectively. All terms in Eq. (1) are expressed as median ± range, and standard rules for error propagation were applied to determine *OD*_*diff*_. Relying on the preliminarily performed FE analysis, estimated values of *OD*_*diff*_ were related to the average shear stress which the specific ROI was exposed to during the flow-exposed culture at the given flow rate. Note that the same CFD model run to design the millifluidic chamber was used to this purpose, modifying the geometry to include the presence of the polyester strip onto the bottom. A schematic of the rationale implemented to associate corresponding *OD*_*diff*_ and shear stress values is reported in Fig. 2.

After that, statistical analysis was performed. First, the biofilm formation on the whole culture substrate was evaluated by computing the median *OD* of those measured in ROIs of all specimens undergoing the same culture conditions. It was then compared among the static control and specimens exposed to the different flow rates, so as to assess whether and how the application of a shear stimulus and steady-flow culture conditions in general has an overall impact on the amount of adhered biomass. To this purpose, a non-parametric Friedman test with *post hoc* Dunn’s comparisons was implemented. In addition, the statistical correlation between *OD*_*diff*_ and the average shear stress of each ROI was tested to assess the potential dependency of differential biofilm growth on the locally applied shear stress. Specifically, the Spearman correlation coefficient was computed for each type of bacterial culture, and—if a significant correlation emerged—a weighted regression line was also estimated (Fig. 2).

## Results

### Generation of Controlled Shear Stress Profiles on the Millifluidic Chamber Bottom

Figure 3a reports the shear stress field on the bottom of the millifluidic chamber determined through the CFD model, and the corresponding profile along the middle longitudinal line for the two considered flow rates is shown in Fig. 3b as a function of the normalized length of the device. A monotonic increase of the shear stress can be observed in the central region of the chamber, while a sharp gradient of shear stress arises close to both the inlet and outlet channel. This behaviour is associated with the presence of a 3D flow and cannot be predicted through simple analytical models. The model also showed that the chamber design, for the set flow rates, generates shear stresses up to $\sim$ 1.5 mPa.

### Biofilm Formation

**Fig. 4:**
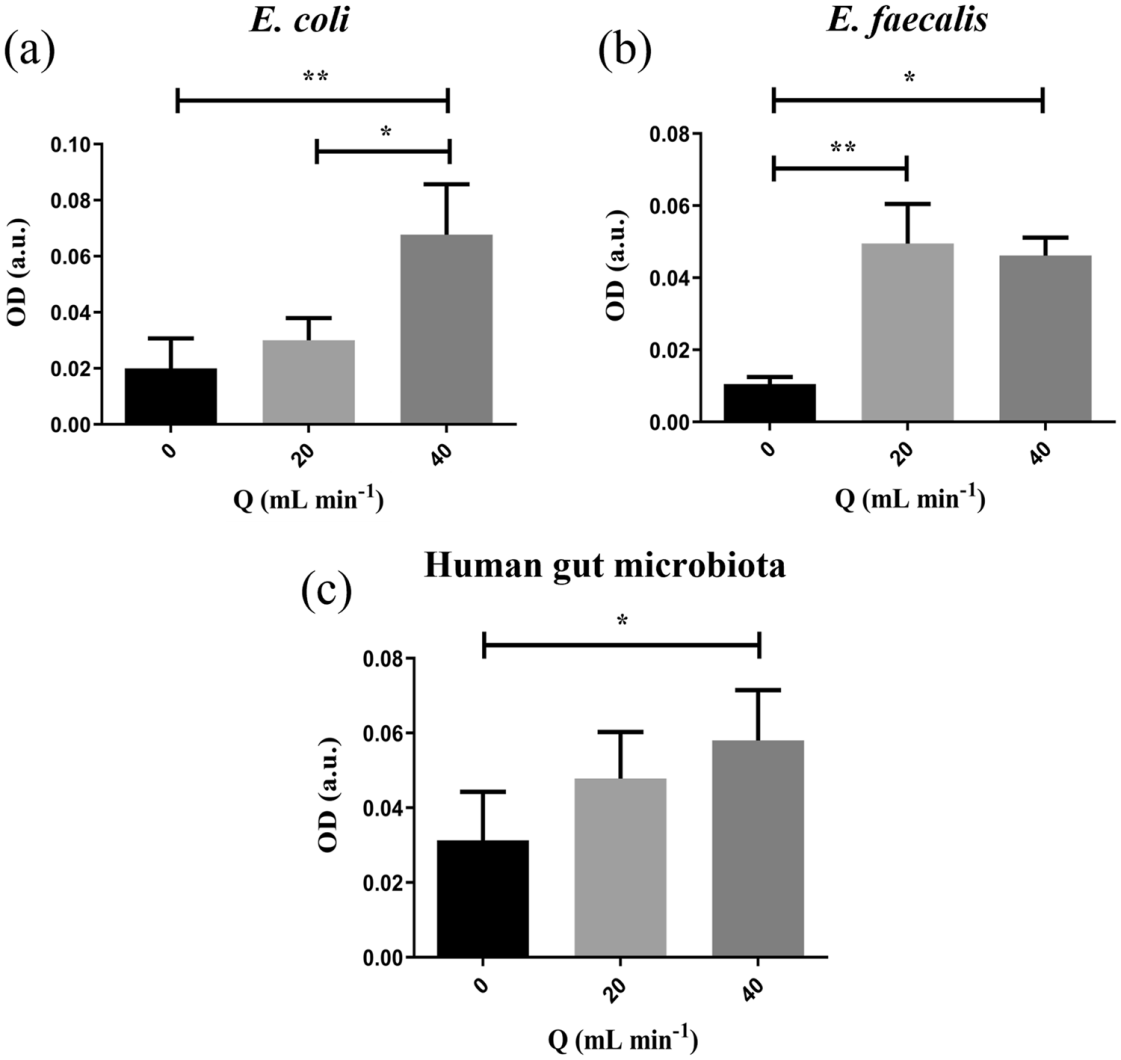
Median OD values of whole microbial specimens after exposure to different flow rates for 8 h (Q = 0 mL min^−1^ refers to static controls). **a** *E. coli*; **b** *E. faecalis*; **c** human gut microbiota. All data are expressed as median ± range (* p < 0.05, ** p < 0.01)

**Fig. 5:**
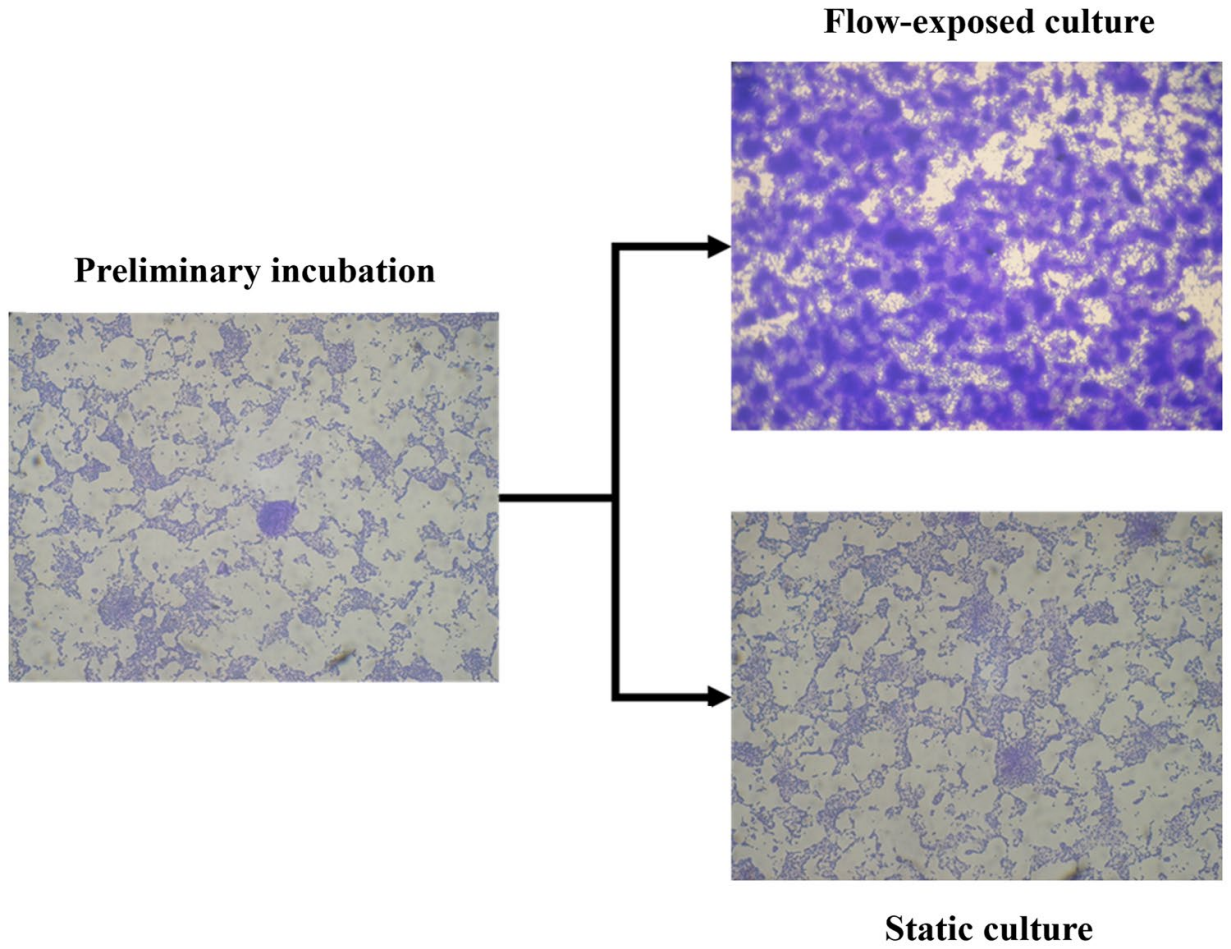
Brightfield images of *E. coli* biofilms acquired after the preliminary incubation within a Petri dish (left), subsequent culturing in static (bottom right) or flow-exposed (*Q* = 40 mL min^−1^, top right) conditions within the millifluidic chamber. All images were acquired with a 20 × objective and refer to the same ROI of the same specimen

Figure 4 summarizes the results of the comparison among specimen exposed to different flow rates and the static control, for both bacterial strains considered (Fig. 4a and b) and for the human gut microbiota (Fig. 4c). The Friedman test highlighted that differences hold among medians (*p* = 0.0012 for *E. coli* and *E. faecalis*, *p* = 0.0162 for the complete microbiota), indicating that, in general, flow exposure affects biomass production. In particular, if compared to corresponding static controls, an increase of the median absorbance and, consequently, of biofilm formation was observed for *E. faecalis* exposed to a flow rate of 20 mL min^−1^ (Fig. 4b), while when the culture medium recirculated at 40 mL min^−1^ all microbial samples enhanced their biomass adhesion onto the substrate. Moreover, no significant differences in biofilm development were reported between the two different flow rates, except for the case of *E. coli* (Fig. 4a). Also, for the sake of visualization, Fig. 5 shows brightfield images of Crystal Violet-stained *E. coli* biofilms acquired before the experiment and after both static culture and flow exposure.

**Fig. 6:**
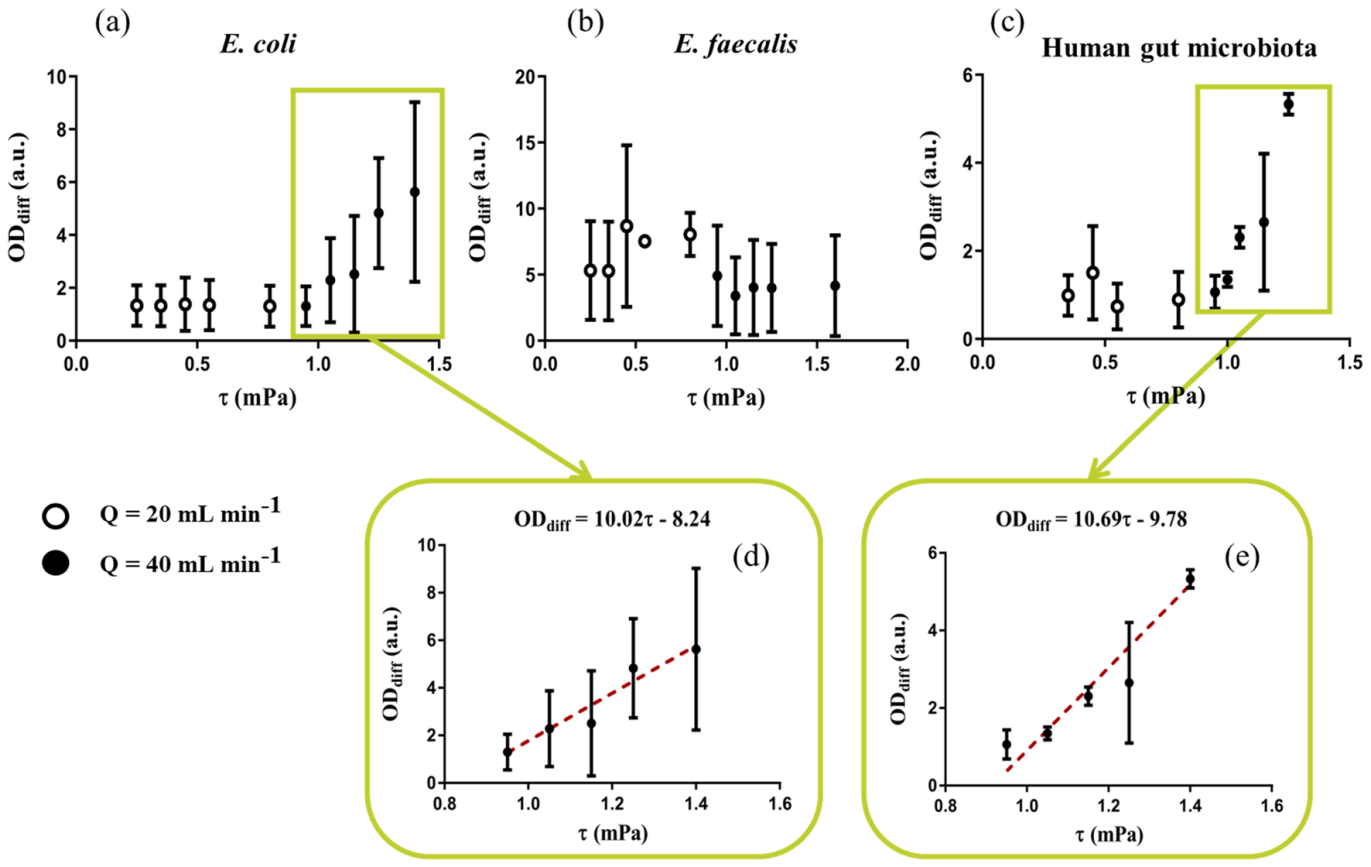
Differential OD as a function of the applied shear stress for: **a** *E. coli*; **b** *E. faecalis*; **c** human gut microbiota. A positive linear correlation emerged in **a** and **c** for shear stress values from 0.95 to 1.40 mPa (corresponding to a flow rate in the millifluidic chamber of *Q* = 40 mL min^−1^). Associated linear fittings are reported as red dashed regression lines in the corresponding insets in **d** and **e**. All datapoints are expressed as median ± range

Figure 6 reports *OD*_*diff*_ measured for different ROIs of the specimen as a function of the average shear stress predicted to be applied on the corresponding ROI by FE analysis. Note that results obtained for both flow rates used (i.e., 20 and 40 mL min^−1^) are plotted on the same graph since the associated shear stress values belong to contiguous intervals. *E. coli* (Fig. 6a) and the human gut microbiota (Fig. 6c) showed an increasing differential biofilm growth along with shear stress between 0.95 mPa and 1.40 mPa. In both cases, this positive trend was corroborated by statistically significant correlations (Spearman correlation coefficients *r* = 0.96 and *r* = 0.95, respectively), and associated linear fittings are reported as insets in Fig. 6d and e (*R*^2^ = 0.9507 for *E. coli* and *R*^2^ = 0.9752 for the complete microbiota). Differently, differential bacterial growth does not relevantly correlate with the applied shear stress magnitude for *E. faecalis* (Fig. 6b). For this bacterial strain, even the lowest levels of investigated stress induced around a 6-fold increase of biomass development with respect to the static control, which is comparable to the differential growth measured for the highest shear stimuli in the other cases.

## Discussion

In this work, a millifluidic chamber allowing to generate controlled shear stress profiles was designed and fabricated as a tool to evaluate how such mechanical stimulus impacts on the development of adherent cellular structures. Unlike similar devices in previous reports from the literature [18, 17, 5], the purposely chosen geometry of our millifluidic chamber enables to expose the biological specimen to a differential stress depending on the spatial position along the principal direction of flow, using a single inlet flow rate. Thanks to proper materials, fabrication techniques and design solutions implemented to realize physical prototypes of the chamber, the surface field of viscous stresses acting on the bottom of the chamber can be reliably predicted by CFD modelling as a function of the set flow rate, ensuring the full, pointwise control of the stress magnitude applied to the cellular sample. As noticeable in Fig. 3, shear stresses comparable with critical values for bacterial biofilm growth estimated by Thomen et al. [16] (i.e., 10 mPa) can be applied to cells or bacteria using this design.

Based on this device, we then proposed an experimental pipeline to assess cellular adhesion depending on shear stress. As a proof of concept, the pipeline was applied to bacterial biofilms of two single strains and of the human gut microbiota. Our findings suggest that applying shear stress to bacterial cultures induces the establishment of denser biofilms (Figs. 4 and 5). Since Crystal Violet stains all components of microbial structures [24, 26], we cannot determine whether the exposure to flow mainly promotes bacterial proliferation rather than the synthesis of extracellular matrix or vice versa. In this regard, contradictory behaviours have been reported, primarily due to the heavy dependency of flow consequences on the specific combination of microbial strain and culture substrate considered [29–33]. Thus, the evidence contributed by the experiments performed in this study only refers to an overall increase of the adhered biomass when shear stress is applied. This response is in line with previous literature reports on bacterial cultures under flow [17, 34–36], and a similar outcome was also obtained for some types of eukaryotic cells in vitro, displaying an increased viability and production of extracellular matrix when exposed to shear forces [37, 7, 38]. In our work, each specimen was cultured for 24 h in static conditions before testing, that is longer than the time reported by Thomen et al. [16], who probed just settled *E. coli* specimens and coherently identified a threshold shear stress above which the establishment of an adherent biofilm is delayed. It is worth to highlight that this pre-culture might have helped microorganisms to better stabilize and create adherent structures. In fact, such incubation time allows microbial adhesion forces to strengthen due to the generation of an extracellular polysaccharide matrix [39], thus improving the resistance to the fluid flow. Nonetheless, as mentioned in the introduction, the effects of shear stress on different microbial species depend on the properties of their external membrane [17]. Different responses of *E. coli* and *E. faecalis* to the application of mechanical stimuli in the same range of magnitude could be interpreted in this light. Flow exposure, indeed, appeared to foster the biofilm formation of *E. faecalis* irrespective of the extent of shear stress that microorganisms undergo (Fig. 6b), since they lack specific appendages—such as type I fimbriae—able to establish stress-dependent adhesion bonds with the culture substrate. These appendages are instead present on the membrane of *E. coli*, which coherently manifested a correlation between the adhered amount of biomass and the applied shear stress (Fig. 6a and d). Such considerations also hold for the human gut microbiota. As a large and heterogeneous population of microorganisms, it could host thousands of species differing in terms of metabolism, dimensions and even membrane properties, so that some of them are promoted to grow and adhere by viscous forces, leading to the trend in Fig. 6c and e.

In conclusion, the reported case study revealed behaviours in line with phenotypic traits of tested microbial specimens, corroborating the robustness of the proposed approach leveraging on the millifluidic chamber. This methodology can be easily generalized, having potential implications towards the design of in vitro platforms which guarantee optimal culture conditions for specific tissue or microbial models.

## Data Availability

All data collected in this work are available upon reasonable request to the corresponding author.

## Abbreviations

3D: Three-dimensional
PDMS: Poly-dimethyl-siloxane
CFD: Computational fluid-dynamics
FE: Finite element
CAD: Computer-aided design
CAM: Computer-aided manufacturing
POM: Poly-oxymethylene
PMMA: Poly-methyl-methacrylate
FDM: Fused deposition modelling
RT: Room temperature
ROI: Region of interest
OD: Optical density
